# Reemergence of Human Monkeypox in Nigeria, 2017

**DOI:** 10.3201/eid2406.180017

**Published:** 2018-06

**Authors:** Adesola Yinka-Ogunleye, Olusola Aruna, Dimie Ogoina, Neni Aworabhi, Womi Eteng, Sikiru Badaru, Amina Mohammed, Jeremiah Agenyi, E.N. Etebu, Tamuno-Wari Numbere, Adolphe Ndoreraho, Eduard Nkunzimana, Yahyah Disu, Mahmood Dalhat, Patrick Nguku, Abdulaziz Mohammed, Muhammad Saleh, Andrea McCollum, Kimberly Wilkins, Ousmane Faye, Amadou Sall, Christian Happi, Nwando Mba, Olubumi Ojo, Chikwe Ihekweazu

**Affiliations:** Nigeria Centre for Disease Control, Abuja, Nigeria (A. Yinka-Ogunleye, O. Aruna, W. Eteng, S. Badaru, Amina Mohammed, J. Agenyi, N. Mba, O. Ojo, C. Ihekweazu);; Measure Evaluation, Abuja (O. Aruna);; Niger Delta University Teaching Hospital/Niger Delta University, Yenagoa, Nigeria (D. Ogoina);; Bayelsa State Ministry of Health, Yenagoa (N. Aworabhi, E.N. Etebu);; Nigeria Field Epidemiology and Laboratory Training Programme, Abuja (T.-W. Numbere, A. Ndoreraho, E. Nkunzimana, Y. Disu);; African Field Epidemiology Network, Abuja (M. Dalhat, P. Nguku);; Africa Centres for Disease Control and Prevention, Addis Ababa, Ethiopia (Abdulaziz Mohammed);; US Centers for Disease Control and Prevention, Atlanta, Georgia, USA (M. Saleh, A. McCollum, K. Wilkins);; Institut Pasteur, Dakar, Senegal (O. Faye, A. Sall); Redeemers University, Ede, Nigeria (C. Happi)

**Keywords:** Human monkeypox, disease, reemergence, Nigeria, zoonoses, viruses

## Abstract

In Nigeria, before 2017 the most recent case of human monkeypox had been reported in 1978. By mid-November 2017, a large outbreak caused by the West African clade resulted in 146 suspected cases and 42 laboratory-confirmed cases from 14 states. Although the source is unknown, multiple sources are suspected.

Human monkeypox is a rare zoonotic infection caused by an orthopoxvirus and characterized by smallpox-like signs and symptoms (*1*). The disease is endemic to the Democratic Republic of the Congo. Reported outbreaks have occurred mainly in rural rainforest areas of the Congo basin and West Africa, caused by the Central and West African clades of the virus, respectively (*1*–*6*). The West African clade is associated with milder disease, fewer deaths, and limited human-to-human transmission. Since 1970, only ≈10 cases in West Africa had been reported; in 2003, a total of 81 cases (41% laboratory confirmed) were reported in the United States (*2*,*7*,*8*). In Nigeria, a case of human monkeypox in a 4-year-old child in the southeastern part of the country was reported in 1971 (*4*,*5*); no more cases in Nigeria had been reported since 1978 (*2*,*6*). We provide a preliminary report of a large outbreak of human monkeypox in Nigeria caused by the West African clade of monkeypox virus in 2017.

On September 22, 2017, the Nigeria Centre for Disease Control (NCDC) was notified of a suspected case of monkeypox; the patient had been admitted to the Niger Delta University Teaching Hospital, Bayelsa State, in the South South region of Nigeria. Outbreak investigations commenced immediately; isolation of the suspected case-patient, laboratory testing, and contact tracing were conducted.

The patient was an 11-year-old boy with an 11-day history of fever, generalized rash, headache, malaise, and sore throat. Physical examination revealed generalized well-circumscribed papulopustular rashes on the trunk, face, palms, and soles of the feet and subsequent umbilication, ulcerations, crusting, and scab formation. The patient had associated oral and nasal mucosal lesions and ulcers and accompanying generalized lymphadenopathy. Similar signs and symptoms, with varying degrees of severity, developed in 5 other family members living in the same household. The index case-patient and 2 of his siblings reported a history of having had contact with a neighbor’s monkey 1 month earlier, but it cannot be ascertained if the monkey was the source of their infection; the monkey had no known history of illness.

After identifying these cases as being suspected monkeypox, the NCDC immediately deployed epidemiologists to Bayelsa State to support detailed outbreak investigations. Health authorities in all states of the country were notified to establish enhanced surveillance based on a standardized case definition. As notification of suspected cases from other states increased, on October 9, 2017, the NCDC activated a national Emergency Operations Centre to coordinate the response to an unusual evolving outbreak. All relevant stakeholders (e.g., ministries of health, agriculture and animal health, and information) were mobilized for a robust response. The NCDC rapidly developed interim guidelines and protocols; disseminated them to all states; and implemented intensive surveillance, public sensitization, community mobilization, and case management accordingly across all states.

Laboratory diagnosis (by real-time PCR, IgM serology, and genomic sequencing) were initially undertaken at Institut Pasteur (Dakar, Senegal), Redeemer’s University Laboratory (Ede, Nigeria), and the US Centers for Disease Control and Prevention (Atlanta, GA, USA). Further diagnostics took place later at the NCDC National Reference Laboratory with technical support from the US Centers for Disease Control and Prevention.

On October 13, 2017, the NCDC received laboratory confirmation of a human monkeypox outbreak in Nigeria. As of November 17, 2017, a total of 146 suspected cases had been reported from 22 of the 36 states in Nigeria (Figure). Of the 134 cases for which samples were available (blood, lesion swab, and crust) collected during the reporting period, 107 were tested, and 42 samples from 14 states were laboratory confirmed as the West African clade of the monkeypox virus (Figure). Most (62%) of the laboratory-confirmed cases were in adults (21–40 years of age; median 30 years of age); the male:female ratio was 2:1. A 46-year-old male patient with confirmed monkeypox and a history of immunosuppressive illness died. For some patients with suspected (but ultimately deemed negative) cases of monkeypox, chickenpox (wild-type virus) was confirmed. Further analysis of the monkeypox-negative samples is ongoing.

**Figure:**
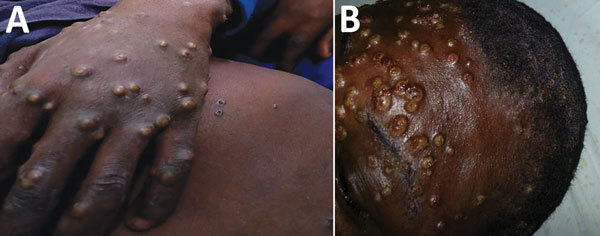
Papulopustular rash on hand (A) and face (B) of patient with monkeypox.

Although detailed epidemiologic investigations to ascertain the source and route of transmission are ongoing, 3 family clusters were found, which might suggest some level of human-to-human transmission in this outbreak. For 1 of the families, the secondary attack rate was 71%. However, most patients had no obvious epidemiologic linkage or person-to-person contact, indicating a probable multiple-source outbreak or possibly previously unrecognized endemic disease. The zoonotic source(s) of the outbreak are currently unknown, and it is unclear what, if any, environmental or ecologic changes might have facilitated its sudden reemergence.

This large outbreak of West Africa clade human monkeypox (*3*,*8*,*9*) mostly affected adults. The NCDC continues response activities and investigations in collaboration with national and international partners. Further findings from our epidemiologic investigations and laboratory diagnostics, including genome sequencing, will add to the existing knowledge of West African monkeypox and help unravel uncertainties in the outbreak.
